# Prevalence of podoconiosis and its associated factors in Gamo zone, Southern Ethiopia, 2021

**DOI:** 10.1186/s13047-022-00517-8

**Published:** 2022-02-10

**Authors:** Tamiru Getachew, Chuchu Churko

**Affiliations:** 1 Department of Medical Anatomy, Arba Minch University, Southern Ethiopia, Ethiopia; 2 Collaborative Research and Training Center for Neglected Tropical Disease, Arba Minch University, Southern Ethiopia, Ethiopia

**Keywords:** Podoconiosis, Disability, Neglected tropical diseases, Ethiopia

## Abstract

**Background:**

Podoconiosis is a chronic non-infectious preventable disease. Though not fatal, it may cause social, economic and physical disability. Ethiopia is projected to bear one-fourth (25%) of the global burden of podoconiosis. Despite its huge economic impact and chronic morbidity and disability, podoconiosis seems to be neglected. Therefore, the aim of this study was to assess the prevalence of podoconiosis and its associated factors in Gamo zone, Southern Ethiopia.

**Methods:**

A community based cross sectional study was conducted among 683 household members. A multistage sampling method was used to select study participants. Binary logistic regression model was fitted to identify factors associated with podoconiosis. Odds ratio with 95% confidence interval was computed to determine the level of significance; in multivariable analysis, variables with a *P* value less than 0.05 were considered as statistically significant.

**Results:**

The prevalence of podoconiosis was 6.2% (95%CI: 4.3–8%). The significantly contributed factors for the prevalence of podoconiosis were wealth index (AOR = 0.249, 95%CI = 0.073–0.845), number of shoes owned (AOR = 6.199, 95% CI = 1.281–29.98), times when individual do not wear shoes (AOR = 2.448, 95%CI = 1.041–5.754), soap utilization during foot washing (AOR = 2.773, 95%CI = 1.210–6.355) and family history of leg swelling (AOR = 4.69, 95%CI = 2.215–9.935).

**Conclusions:**

This study showed that there was significant burden of podoconiosis in the study area. Wealth index, times when individual do not wear shoes, number of shoes owned, soap utilization during foot washing, and family history of leg swelling were significantly associated with podoconiosis. It is recommended to practice secondary prevention which includes regular foot hygiene and wearing shoes, and the use of antiseptic soaks.

## Background

Podoconiosis (endemic non-filarial elephantiasis) is a chronic non-infectious neglected tropical disease (NTD) which affects the lower limb. Although the causes of podoconiosis is not fully understood, current evidence suggest that it is caused by longstanding exposure to red clay soil of volcanic origin [1]. Mineral particles, absorbed through the skin of the foot, are taken up into macrophages in the lower limb lymphatics and are thought to induce an inflammatory response in the lymphatic vessels, leading to fibrosis and obstruction of the vessel lumen [2].

The early symptoms of podoconiosis include itching of the skin of the forefoot and a burning sensation in the foot and lower leg [3]. Later on it develops persistent swelling of the foot which starts from the dorsum of foot and gradually progress to the lower leg. The swelling is bilateral which is asymmetric and usually limited below the knees with mossy and nodular changes to the skin [4–6].

Podoconiosis is classified into five stages based on clinical characteristics; swelling limited below ankle and reversible overnight (stage I), swelling is not reversible, and when bumps and knobs are present and they remain below the level of the ankle (stage II), bumps and knobs are found above the level of the ankle (stage III), swelling extends above knee and not completely reversible overnight (stage IV) and joint fixation as a result of surrounding soft tissue overgrowth (stage V) [7].

Currently, podoconiosis does not have specific treatment, but primary prevention consist of avoidance of prolonged contact between the skin and irritant soils by robust footwear or covering of floor surfaces in areas of irritant soil, training in foot hygiene like washing the legs daily with soap and water, using antiseptics and emollients [8].

Although, podoconiosis is not fatal, affected individuals may show spoiled appearance of legs and the quality of life may be reduced [9, 10]. Clinically, most patients acquire repeated infections of bacterial and fungal nature in the affected leg(s) necessitating extra medical attention. In addition the patients may experience acute adenolymphangitis (ALA) several times a year. It has been estimated that they lose an average of one month of economic activity every year due to morbidity [11]. In addition, people with podoconiosis may be stigmatized by being excluded from school, local meetings, churches, and barred from marriage with unaffected individuals [12].

The disease is widespread in tropical Africa, Central America and North India [13]. Evidence showed that one–sixth of the world’s population, mostly in developing countries, is infected with one or more of the NTD (neglected tropical diseases). The World Health Organization (WHO) has identified seventeen NTDs for control and elimination at the global level [14] and among these diseases, eight were identified, including podoconiosis as priority in Ethiopia with a range of endemicity across the regions [15].

The national average prevalence was 4% with the highest prevalence in SNNPR(Southern Nations Nationality and People Regional) (8.3%) followed by Oromia (4%) and Amhara (3.9%) regional states. Nationally, it is estimated that there are 1.56 million cases of podoconiosis and there were 345 districts with the prevalence of disease greater than 1% [16].

Despite its huge economic impact and chronic morbidity and disability [10, 11, 17], podoconiosis seems to be neglected and not considered as public health problem in the study area. This study was the first in the area for assessing the prevalence and associated factors of podoconiosis.

## Methods and materials

### Study setting and period

This study was conducted in in Gamo zone, which is found in SNNPR, from January to February 2021. A community based cross sectional study was employed to assess prevalence of podoconiosis and its associated factors. Gamo zone is one of the administrative zones in southern Ethiopia. It is bordered by Wolayta, Dawro, and Gofa zones in the North, Lake Abaya in the northeast, and Amaro special woreda and Dirashe special woreda in the southeast, and South Omo in the southwest. The administrative center of the Gamo zone is Arba Minch town. Gamo zone has one administrative town and 18 woredas. Total population of the former Gamo gofa zone as to 2007 census was, 1, 593, 104, among which 49.8% (793,322) were males and 49.2% (799, 782) were females. About,1,435,658(90.1%) were rural dwellers [18].

### Study design and eligibility criteria

A community based cross-sectional study design was employed. Those individuals whose age were 15 years old and above were eligible for this study and those individuals who were mentally handicapped and critically sick were excluded.

### Population

All individuals living in Gamo zone whose age was 15 years and above were the source population. An individual with age 15 and above in purposively selected districts and who fulfilled the inclusion criteria were the study population.

### Sample size determination and sampling procedure

The sample size was calculated using one population proportion formula of cross sectional study by considering the following assumptions: P (Prevalence of podoconiosis in Wolayta zone, Sodo zuria district 5.4% [19], **α (**level of significance) **=** 5%, The Z-value at 95% CI and 5% **α** = ±1.96 (two tailed), Margin of error (W) = 2.5%, power of study =80%. Then by using design effect of 2 and adding 10% non-response rate, the final sample size was 690 individuals from randomly selected household were included. A multi stage sampling method was applied. From administrative districts in Gamo zone, purposefully three districts were selected based on expert opinion for the presence of podoconiosis cases. After getting list of kebeles (smallest administrative units) from selected districts, couples of kebele per districts were selected by lottery method. Then, we took households list from selected kebele and feed to computer program Microsoft excel to select final participant households using computer driven random number by considering probability proportional to size **(**PPS**)** of households in the kebele.

Systematic selection of the households was done depending on the total number of households to the sample households required from each kebele by dividing the number of households in each kebele by sample size in that kebele. One participant was randomly selected from each household. On the other hand, in case there was no eligible subject in the selected household, the next immediate neighbor’s household with eligible study subject was included in the study.

### Study variables

#### Dependent variables

Presence or absence of podoconiosis**.**

#### Independent variables

Socidemographic variables: Age, sex, gender, educational level, marital status, ethnicity, religion, occupation, number of years lived in area, wealth index.

Shoe wearing and personal hygiene: age at the first shoe wearing, number of shoes owned, frequency of shoe wearing, utilization of soap during foot washing, average distance from water source to home.

### Data collection procedure and collection instrument

Data was collected using interviewer administered structured questionnaire and observational check list. The questionnaire and observation check list were adapted and prepared from related literatures with modification to local context. It was prepared by English language and then translated to Amharic language and back to English language to ensure consistence. The questionnaire was sub-sectioned thematically to include: socidemographic characteristics, foot wearing and personal hygiene characteristics, knowledge about podoconiosis causes and prevention. Observational check list was utilized for clinical examination which contained diagnosis and staging of podoconiosis, assessment of ALA and measurement of leg circumference. Eight BSc nurses and health officers who had experience in diagnosing and treating the disease were recruited for data collection and three master degree holder were selected for supervision and trained for three days.

The researchers trained the nurses and the health officer on: the nature, etiology, treatment and prevention of podoconiosis, clinical features that differentiate podoconiosis from other diseases such as leprosy and filarial elephantiasis, assessment of ALA, assessment of presence of open wounds and mossy lesions.

Practical training was provided on clinical diagnosis of podoconiosis based on clinical algorithm for the diagnosis of podoconiosis for endemic areas [20], disease staging using a recently developed five level podoconiosis staging system [7] and the procedure for measuring of leg circumference [21].

The health extension workers and village chairpersons guided the interviewers during house-to-house visits. The nurses registered households that included a podoconiosis patient, administered a structured questionnaire to these patients, and conducted a physical examination of the legs and feet of patients. The legs and feet were assessed for clinical stage of disease. The largest circumference of the leg between the levels of the ankle and knee was measured using a tape to a precision level of the nearest centimeter. The study participants were asked to stand and the tape placed around the calf at the widest part between the lateral side of the ankle and knee as anatomical landmarks, and was ensured that the tape was horizontal around the calf and moved the tape up and down to locate the maximum circumference. Each data collector measured three times to the nearest 0.1 cm and finally the mean of three measurements was taken.

### Operational definitions

#### A person with podoconiosis

An individual who has history of burning sensation in the feet when the swelling started; visible swelling that started at the feet and progressed upwards and with no known clinical signs or symptoms of leprosy or lymphatic filariasis [22].

#### ALA

A reddish hot, swollen leg with a painful groin [23].

#### Leg circumference

The largest circumference between the level of the ankle and the knee measured using a tape, to a precision level of the nearest centimeter [7].

#### Mossy lesions

Papillomatous horny lesions giving the skin a rough appearance [22].

#### Criticality sick

patients who could not respond to the interview due to medical illness [24].

### Data quality assurance

Data cleaning was performed to assess completeness, consistence, outliers and missed values. Training was given for data collectors and supervisors. Pretest was conducted on 5% households outside of study kebele, necessary correction was made and questionnaire was further modified after a pretest. By taking 5% of the collected data randomly, the consistence was cross-checked based on the household code. If any error was identified during review, it would have been corrected accordingly by supervisors and investigators. Maximum effort was made to minimize inter observer bias during diagnosing presence of podoconiosis and measuring of leg circumference. Each data collectors measured the leg circumference three times and they took the average value of the three measurements to address intra-rater variation. During training session we have given practical training on leg circumference using pictures and animated videos to control inter-rater variation.

### Data processing and analysis

After checking completeness of the collected data, data was transferred in to Microsoft excel for Windows 2010 and further transferred to SPSS software version 20 to make ready for data cleaning and analysis. Descriptive statistics was performed and presented by Mean (±SD) for continuous normally distributed variables. Frequency and percentage, cross tabulation were performed for categorical predictors. Principal component analysis was performed to generate a wealth index. We used quintiles to categorize the wealth index. Binary logistic regression analysis was performed to see the independent effect of predictors on prevalence of podoconiosis. Bivariate logistic regression analysis was conducted to select potential candidates for the next step using *P*-value criteria of ≤0.25.

Multivariable logistic regression analysis was performed to identify the independent effect of predictor’s after controlling for potential confounders. Step wise back ward elimination model building procedure was done and model was compared by likely hood ratio test. Interaction and cofounder was tested and cutoff point was beta change greater than 20%.

Multi colinearity was checked using variance inflation factor (VIF) and cutoff point was mean VIF > 10 to have significant colinearity among predictors. Overall model fitness was checked by Hosmer and Leme show chi-square test; associations between prevalence of podoconiosis and predictors was summarized by using adjusted odds ratio and statistical significances was tested by Wald chi-square test at 95% CI and 5% of α.

## Results

### Socio-demographic characteristics of participants

Out of 690 participants, 683 households were interviewed making the response rate 99%. Of 683 participants, 41% were male and 59% were female. The mean age (SD+) of the participant was 45.33 + 14.44. On average, the respondents had lived in the study are for 32.19+ 19.94 years. Half of the study participants (*n* = 344, 50.4%) had no formal education (Table 1).

**Table 1:** Socio-demographic characteristics of participants in Gamo Zone, Southern Ethiopia, 2021 (n-683). (Others* include –NGO employee (0.1%); daily laborer (2.8%); jobless (1.3%)

Variable	Category	Frequency	Percentage
Age group	15–24	46	6.7
	25–34	109	16.0
	35–44	167	24.5
	45–54	170	24.9
	> 55	191	28.0
Years lived in area	3–10 years	26	3.8
	11–18 years	37	5.4
	19–26 years	78	11.4
	27–36 years	106	15.5
	37–46 years	162	23.7
	> 47	274	40.1
Gender	Male	280	41
	Female	403	59
Education level	No formal education	344	50.4
	Able to read and write	153	22.4
	Grade 1–8	94	13.8
	Grade 9–12	55	8.1
	More than secondary	37	5.4
Marital status	Never married	69	10.1
	Married	564	82.6
	Widowed	39	5.7
	Divorced	11	1.6
Ethnicity	Gamo	674	98.7
	Amhara	8	1.2
	Wolayta	1	1
Occupation	Farmer	424	62.1
	Merchant	44	6.4
	Student	39	5.7
	Housewife	115	16.8
	Government employee	32	4.7
	Other*	29	4.2
Religion	Protestant	295	43.2
	Orthodox	387	56.7
	Muslim	1	.1
Monthly income	< 500	241	35.3
	> = 500	442	64.7
Wealth index quintile	Lowest	134	19.6
	Second	145	21.2
	Middle	200	29.3
	Fourth	18	2.6
	Highest	186	27.2

### Prevalence and clinical feature of podoconiosis

**Fig. 1:**
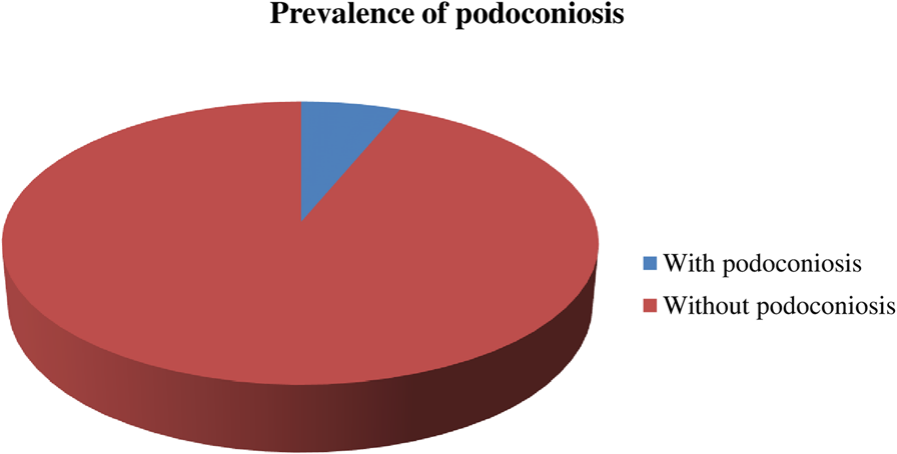
Prevalence of podoconiosis in Gamo zone, Southern Ethiopia, 2021

**Fig. 2:**
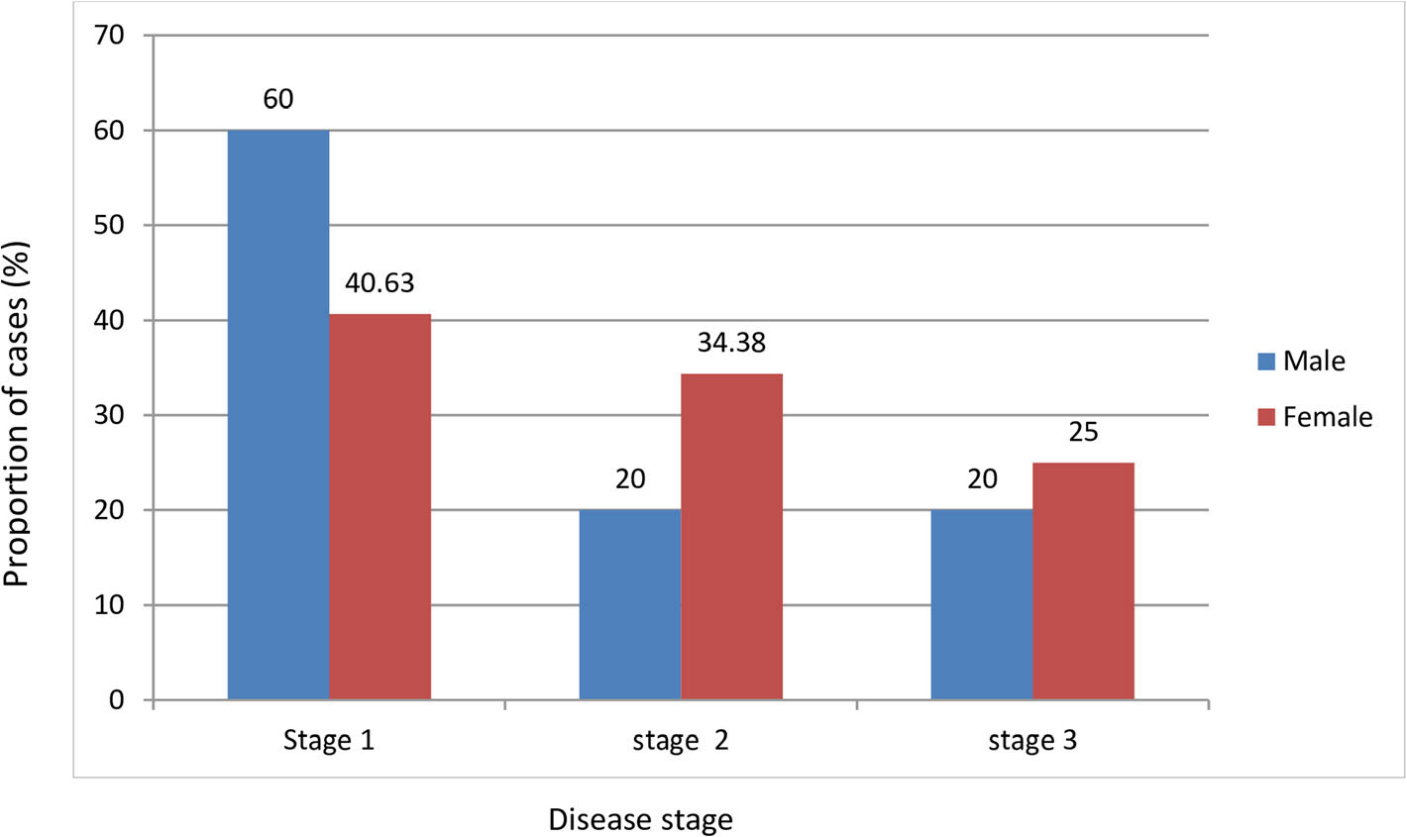
Clinical stages of podoconiosis among female and male patient in Gamo zone, Southern Ethiopia, 2021

The prevalence of podoconiosis in this study was found to be 6.2% (95% CI: 4.3–8%) (Fig. 1). The proportion among females was (*n* = 32, 76.2%) and female to male ratio was 3.2:1. The average duration of illness between time of onset and time of interview was found to be 10.26 + 8.57 days. The highest prevalence of podoconiosis, (*n* = 12, 28.6%), was observed among age groups > 55 years. Mossy lesions and open wounds were observed in (*n* = 12, 28.6%) and (*n* = 5, 11.9%) of patients, respectively (Table 2). Among clinically diagnosed patients, (*n* = 19, 46.6%) were identified in stage one, (*n* = 13, 31.03%) in sage two and (*n* = 10, 22.4%) stage three. More than half, (*n* = 6, 60%) of affected male patients were in stage one (Fig. 2).

**Table 2:** Clinical feature of podoconiosis and ALA in Gamo Zone, Southern Ethiopia, 2021

Variable	Category	Frequency	Percent
Sex	Male	10	23.8
	Female	32	76.2
Age category	15–24	4	9.5
	25–34	7	16.6
	35–44	9	21.4
	45–54	10	23.8
	> 55	12	28.6
Staging of the diseases	Stage one	19	45.2
	Stage two	13	31
	Stage three	10	23.8
Sought treatment	Yes	12	28.6
	No	30	71.4
Treatment site	Traditional healers	5	41.7
	Health institution	7	58.3
Mossy lesion	Present	12	28.6
	Absent	30	71.4
Open wound	Present	5	11.9
	Absent	37	88.1
Right leg circumference	< 21	6	13.9
	21–25	7	16.7
	26–36	12	28.6
	37–47	7	16.7
	> 47	10	23.8
Left leg circumference	< 21	5	11.9
	21–25	9	21.4
	26–36	10	23.8
	37–47	5	11.9
	> 47	13	30.9
ALA at the time of interview	Present	27	16.7
	Absent	35	83.3
Last time the patient had ALA	Last 2 weeks	7	16.7
	Last 1 month	6	14.3
	Last 6 months	11	26.2
	Beyond 1 year	18	42.9
Sought treatment for ALA	Yes	12	71.4
	No	30	28.6
Season when symptoms of ALA get worse	Rainy and wet season	19	45.2
	Hot and dry season	13	31
	No specific season	10	23.8
Coping mechanism for ALA(n = 42)	Using antibiotics	20	47.6
	Washing feet	22	52.4
	Stay in bed	25	59.5
	Resort to less exertion	17	40.5
	Using traditional herbs	11	26.2
Precipitating factors	Long walks	19	45.2
	Mitch	18	42.9
	Laborious work	20	47.6
	Dust	24	57.1

### ALA and coping strategies among podoconiosis patients

On history and clinical examination about (*n* = 7, 16.7%) patients had ALA at the time of interview. About (*n* = 18, 42.9%) had episode of ALA in previous one year and (*n* = 7, 16.7%) had ALA two weeks prior to the date of interview. The coping strategies adopted to reduce the morbidity during episodes of ALA were (*n* = 25, 59.5%), stay in bed, (*n* = 20, 38.1%) using antibiotics, (*n* = 17, 40.5%) resort to less exertion and (*n* = 11, 26.2%) using traditional herbs [Table 2].

### Shoe wearing and personal hygiene characteristics of the respondents

All respondents were asked about their experience and attitudes towards footwear and personal hygiene, which are central to prevent, treat and control disease progression**.** The mean age of the participants (+SD) when they had their first shoes wearing was 8.36 + 7.26 years old. Only about (*n* = 70, 10.2%) of the participant did wear shoes during farming work and (*n* = 425, 62.2%) never walks bare foot. Regarding the frequency of feet washing, about (*n* = 656, 96%) wash their feet daily. Nearly two fifth of the participants (*n* = 270, 39.5%) did not use soap during feet washing. On observation more than three fourth (*n* = 513, 75.1%) of participants had clean and intact feet, (*n* = 139, 20.4% and (*n* = 31, 4.5% of participants had dirty and both dirty and cracked feet respectively (Table 3).

**Table 3:** Foot wearing and personal hygiene related characteristics of the participants, Gamo Zone, Southern Ethiopia, 2021

Characteristics	Category	Frequency	Percentage
Average age of first shoes wearing	Mean (SD)	8.36 + 7.26 years	
Number of pairs of shoes owned	One pair	167	24.5
	Two pairs	269	39.4
	Three pairs	171	25.0
	More than three pairs	76	11.1
Type of shoe worn during the interview	Not footwear	53	7.8
	Hard plastics	354	51.8
	Canavas	223	32.7
	Open sandal	53	7.8
Times when individuals do not wear shoes	During farming work	70	10.2
	During non-farming work	49	7.2
	at home	139	20.4
	never walks bare foot	425	62.2
Feet cleanliness at the time of interview	Intact feet	513	75.1
	Dirty feet	139	20.4
	Both dirty and cracked feet	31	4.5
Frequency of foot washing	Daily	656	96.0
	Two to three times per week	27	4.0
Use soap for washing	Yes	413	60.5
	No	270	39.5
Source of water	Pipe	490	71.7
	Borehole or well	96	14.1
	River or stream	78	11.4
	Pond or stagnant	19	2.8
Average distance from water source to home	Near home (less than 30 min)	329	48.2
	Far away from home (more than 30 min)	354	51.8
Types of floor in your household	Earth	517	75.7
	Non-earth	166	24.3
Types of toilet facility	Pit latrine without slab/open pit	511	74.8
	Pit latrine with slab	139	20.4
	No facility/bush field	33	4.8

### Characteristics of knowledge related aspects

Out of 683 participants, only (*n* = 232, 34%) were heard about podoconiosis. From those who heard about the disease, nearly half (*n* = 84, 46.4%) of the study participant reported that podoconiosis could be prevented by wearing protective shoes whereas 15.9% said it is preventable by not marrying affected person. More than three fourth of the participants (*n* = 181, 78%) said that podoconiosis is preventable (Table 4).

**Table 4:** Knowledge related aspects among people with and without podoconiosis in Gamo zone, Southern Ethiopia, 2021

Variables	Category	Podoconiosis status	
		Yes	No
Heard of podoconiosis	Yes	15(35.7%)	211(32.9%)
	No	27(64.3%)	430(67.1%)
Cause of podoconiosis	Soil particles	7(25%)	68(33.3%)
	Poverty	6(21.4%)	45(22.1%)
	Malnutrition	7(25%)	30(14.7%)
	Devil spirit	4(14.3%)	15(7.4%)
	Hereditary	4(14.3%)	14(6.9%)
	Snake bite	0	32(15.7%)
Podonconsis is preventable	Yes	22(78.6%)	159(77.9%)
	No	6(21.4%)	45(22.1%)
Methods of podonconsis prevention	Wearing protective shoes	11(50%)	73(45.9%)
	Washing feet frequently	4(18.2%)	52(32.7%)
	Avoiding marriage with affected person	4(18.2%)	25(15.7%)
	Avoid with working cold air	3(13.6%)	9(5.7%)
Podonconsis curable	Yes	18(64.3%)	108(52.9%)
	No	10(35.7%)	96(47.1%)
Podonconsis transmits from person to person	Yes	12(42.9%)	73(35.8%)
	No	16(57.1%)	131(64.2%)

### Factors associated with podoconiosis

Gender, marital status, wealth index, number of shoes owned, times when individual do not wear shoes, soap utilization during foot washing, family history of leg swelling, feet cleanness at time of interview, distance of water source from home, time taken to health facility were found associated with podoconiosis in bivariate analysis using *p*-values< 0.25 cutoff.

In multivariable logistic regression analysis wealth index, number of shoes owned, times when individual do not wear shoes, soap utilization during foot washing and family history of leg swelling were significantly associated with podoconiosis.

In this study, those participants who had lowest wealth index were less likely to develop podoconiosis than those who had highest wealth index (AOR = 0.249, 95%CI =0.073, 0.845, *P* < 0.026). Podoconiosis among participants who did not wear shoes at home were about 2.5 times higher than as compared to those who never walked bare foot (AOR = 2.448, CI = 1.041–5.754, *P* = 0.04). The Odds of developing podoconiosis among those who did not use soap during foot washing nearly three times higher compared to those who did use soap during foot washing (AOR = 2.77, 95%CI = 1.210–6.355, *p* = 0.016). Moreover, the odds of developing podoconiosis among those who had one pair of shoes were six times higher than to those who had more than three pairs of shoes.(AOR = 6.19,95% CI = 1.281–29.98,*P* = 0.023). The participants who had a family history of leg swelling was about five times more likely to develop podoconiosis than who had not family history of leg swelling (AOR = 4.669, 95%CI = 2.215–9.935, *P* < 0.001) (Table 5).

**Table 5:** Factors associated with podoconiosis among people who are living in Gamo zone, Southern Ethiopia, 2021

Variable	Category	Podoconiosis		COR(95%CI)	AOR(95%CI)	***p***-value
		Yes	No			
Gender	Male	10	270	1		
	Female	32	371	0.429(0.208–0.889)	0.525 0.240, 1.145	0.105
Marital status	Never married	7	62	1	1	
	Married	32	532	0.533(226, 1.258)	0.487(0.153,1.547)	0.223
	Widowed	2	37	0.479(094,2.427)	0.194(0.029,1.297)	0.091
	Divorced	1	10	0.886(098,7.987)	0.861 0.077,9.615	0.903
Wealth index (quintiles)	Lowest	5	129	0.617(0.209, 0.381)	0.249 (0.073, 0.845)	0.026*
	Second	6	139	0.687(0.248,0.470)	0.607(0 .204, 1.807)	0.370
	Middle	17	183	1.478(0.673, 0.330)	1.416(0.594, 3.377)	0.433
	Fourth	3	15	3.182(0.800, 0.100)	2.765(0 .578, 13.231)	0.203
	Highest	11	175	1	1	
Number of shoes owned	One pair	20	147	7.45(1.72–32.16)	6.199(1.281, 29.98)	0.023*
	Two pairs	9	260	0.7(0.133–3.68)	1.678(0.325,8.669)	0.537
	Three pairs	11	160	1.57(0.32–7.79)	3.508(0.693,17.76)	0.129
	More than three pairs	2	74	1	1	
Types of foot wear at time of interview	not footwear	7	46	1.461(0.433, 4.932)	0.977(0.237,4.023)	0.975
	hard plastics	22	332	0.636(0.230–1.759)	0.859(0.251,2.933)	0.808
	canavas	8	215	0.357(0.112–1.140)	0.421(0.106,1.675)	0.219
	open sandal	5	46	1		
Times when individual do not wear shoes	During farming work	7	63	2.374(0.959,5.877)	1.461(0.499,4.280)	0.490
	During non-farming work	3	46	1.394(0.397–4.890)	1.092(0.268,4.452)	0.903
	at home	13	126	2.205(1.059–4.590)	2.448(1.041,5.754)	0.040*
	never walks bare foot	19	406	1	1	
Soap using during foot washing	Yes	20	393	1		
	No	22	248	1.74(0.932,3.26)	2.773(1.210, 6.355)	0.016*
Family history of leg swelling	yes	18	89	4.652 (2.426,8.918)	4.69 (2.215,9.935)	0.000*
	No	24	552	1		
Feet cleanness at time of interview	Clean and intact feet	26	487	1	1	
	Dirty feet	14	125	2.09(1.064, 4.136)	0.353 (0.996,5.557)	0.051
	Both dirty and cracked feet	2	29	1.29(0.292,5.710)	1.741(0.324,9.362)	0.518
Distance of water source	Near home (less than 30 min)			1	1	
	More than 30 min			0.681(0.362–1.27)	0.705(0.323 1.541)	0.381
Time taken to heath facility	< 1.5 h	21	416	1		
	> 1.5 h	21	225	1.84(0.98,3.5)	1.409(0.689,2.87)	0.347

## Discussion

This is the first community-based study attempted to assess the prevalence and factors associated with podoconiosis in Gamo zone, Southern Ethiopia. The present study showed that podoconiosis is a problem of public health importance.

The prevalence of podoconiosis in this study was 6.2% (95% CI: 4.3–8%).This findings is in line with the study conducted in Dano district, Ethiopia (6.3%) [25]. This similarity might be due to the fact that the two populations have similar socio-demographic and life styles. On the contrary, this prevalence is high when compared to studies from Sodo zuria, southern Ethiopia (5.4%) [19], Guliso district, west Ethiopia (2.8%) [23], Wayu district, Ethiopia, 3.05% [22] and Bedela Zuria of west Ethiopia, 5.6% [26]. Similarly, our finding was high as compared to studies from African countries like Kenya 3.4% [27] and Cameron 0.5% [28]. This difference might be due to podoconiosis prevention measures have been conducted more often than the current study area. In contrast, the finding of this study was low as compared to a study conducted in Midakegn district, Ethiopia (7.4%) [29]. This discrepancy might be due the difference in intervention provision, accessibility of water and increasing awareness of shoes wearing practice of the participants.

A study conducted by Alemtsehay and his colleagues reported that a participant who had low wealth index was likely affected by podoconiosis than participants who had high wealth index [23, 30]. Surprisingly, in our study the participants who had low wealth index were less likely affected by podoconiosis than participants who had highest wealth index. The possible reason could be participants who had high wealth index might be genetically more susceptible to develop podoconiosis than participants who had low wealth index. However, this variable needs to be studied further.

The current study showed that shoes wearing practice was associated with developing of podoconiosis. The participants who did not wear shoes at home were about 2.5 times higher chance of being affected by podoconiosis as compared to those who never walk bare foot (AOR = 2.448,CI: 1.041–5.754). This agreed with the previous studies [25, 27]. This is due to as majority of the participant’s the floor of the house made from earth this soil particles enter in to the skin of uncovered feet. A long term exposure to soil particles stimulates a provocative reaction in the lymphatic system which causes thickening and obstruction of lymphatic system [31].

Podoconiosis among individuals who had one pair of shoes were six times significantly higher than those who had more than three pairs of shoes (AOR = 6.19, 95% CI: 1.281–29.98). This is because having more shoes will improve the frequency of shoes wearing practice. This will reduce exposure to the irritant soil which is believed to be a cause of podoconiosis.

The odds of developing podoconiosis among those who did not use soap during foot washing were three times higher than those who utilized soap (AOR = 2.77,95%CI: 1.210–6.355). This is consistent with the study conducted by Dejene and his colleagues in central Ethiopia [25] and in Sodo zuria, Southern Ethiopia [30]. This is because washing foot with soap removes soil particles that triggers the diseases process and prevent infection.

A study conducted on a topical model for gene –environment interaction showed a strong genetic component, the sibling of an affected person is at five times increased risk of developing podoconiosis when compared with a person in the general population [13]. Similarly, in our study the participants who had a family history of leg welling is about five times more likely to develop podoconiosis than who had no family history of leg swelling. (AOR = 4.67, 95%CI (2.22–9.94). This is might be due to the effect of genes in the development of podoconiosis; this result still requires further genetic analysis.

In the present study majority of patients were in the early stage of podoconiosis (Stage I and Stage II). This agreed with a study conducted in Dana district and Jeldu district west shoea, Ethiopia [25, 32]. Since most cases of podoconiosis in present study were in early stage of disease, secondary prevention is potentially helps to control the diseases progression. A study conducted in southern Ethiopia showed that a reduction in leg circumference and an improvement in clinical stage were observed following the use of simple lymphedema management method including regular wearing of shoes, frequent washing of foot with soap and water and utilizing bandage [33].

### Strength and limitation of the study

As strength this study was the first study in the area to assess the prevalence and associated factors of podoconiosis. The study is not without limitation. Firstly, it is likely to face issues of non-response bias due to stigma families may hide affected members. We attempted to minimize undercounting by using health extension workers as data collectors. They are familiar, trusted community members who know most families in their kebeles. Secondly, we used a clinical algorithm to differentiate podoconiosis from other disease causing leg swelling. If serological test was used, it would have helped to exclude it from lymphatic filariasis. However, given that the study area is found at high elevation above sea level, transmission of filarial is extremely unlikely. Thirdly, even if we estimated the prevalence of podoconiosis we could not able to generalize the findings to Gamo zone as the districts were selected purposively.

## Conclusion and recommendation

This study reported a high prevalence of podoconiosis and its associated factors in the study area. Number of shoes owned, wealth index, times when individual do not wear shoes, family history of leg swelling, soap utilization during foot washing were found associated with development of podoconiosis.

As majority of the patient were in early stage of the disease. It is recommended to practice secondary prevention which includes regular foot hygiene and wearing shoes. Health education shall be given for increasing protective shoe wearing practice, improving personal hygiene and treatment seeking behavior. Governmental and non-governmental organization should work by integrating existing health programs addressing water and sanitation and neglected tropical disease. Podoconiosis treatment and rehabilitation center shall be established.

## Data Availability

The datasets for the current study are not publicly available but are available upon reasonable request by emailing the corresponding author.

## Abbreviation

ALA: Acute adenolymphangitis
NTD: Neglected Tropical Disease
SNNPR: Southern Nations Nationality and People Regional State
VIF: Variance Inflation Factor
WHO: World Health Organization
